# The effectiveness of motherwort injection in preventing postabortion hemorrhage after induced abortion: A protocol for systematic review and meta-analysis

**DOI:** 10.1097/MD.0000000000032935

**Published:** 2023-02-17

**Authors:** Yanjie Xiang, Xiaohan Wang, Yongqian Gong, Jianfeng Xiang

**Affiliations:** a Center for Reproductive Medicine, Xinhua Hospital Affiliated to Shanghai Jiao Tong University School of Medicine, Shanghai, P.R. China; b Department of Obstetrics, Rizhao Hospital of Traditional Chinese Medicine, Shandong, P.R. China; c Department of Otorhinolaryngology, The First Affiliated Hospital of Hengyang Medical School, University of South China, Hunan, P.R. China; d Department of Interventional Oncology, Renji Hospital, Shanghai Jiao Tong University School of Medicine, Shanghai, P.R. China.

**Keywords:** abortion, hemorrhage, meta-analysis, motherwort, protocol, review

## Abstract

Unintended pregnancy is a problem that women encounter throughout their reproductive age. Excessive and prolonged uterine bleeding is one of the most common and critical adverse reactions of induced abortion, for it increases the risk of anemia and intrauterine infection. To provide reliable clinical evidence, we performed a protocol for systematic review and meta-analysis to evaluate the hemostatic effect of motherwort in postabortion.

**Methods:**

This review protocol has been registered in the international prospective register of systematic reviews. The statement of Preferred Reporting Items for Systematic Review and Meta-Analysis Protocols will be used as guidelines for reporting present review protocol. Original clinical randomized controlled trials assessing the beneficial effects and safety of motherwort on induced abortion will be included. Databases searched include China National Knowledge Infrastructure, Chinese Scientific Journals Database, Wanfang Database, China Biological Medicine Database, PubMed, and EMBASE Database and Cochrane Central Register of Controlled Trials. Cochrane collaboration tool is used to assess the risk of bias of included randomized controlled trials. All calculations are carried out with Stata 11.0 (The Cochrane Collaboration, Oxford, United Kingdom).

**Results:**

This systematic review and meta-analysis will provide a detailed summary of the current evidence related to the efficacy of motherwort injection preventing postabortion hemorrhage after induced abortion.

**Conclusion:**

This evidence will be useful to practitioners, patients, and health policy-makers regarding the use of motherwort injection in induced abortion.

## 1. Introduction

More than 300 thousand maternal deaths occur every year; most of these are preventable.^[1]^ Ninety-nine percent of these maternal deaths occur in developing countries. Recent studies have shown that the mortality risk from an induced abortion is 0.7 per 100,000.^[2]^ Minor complications have dropped to an estimated 8 per 1000 abortions by 1990, and major complications have decreased to 0.7 per 1000 induced abortions.^[3]^ Both hemorrhage and anesthetic complications have been the leading causes of abortion-related mortality.^[4]^ Hemorrhage is the leading cause of mortality during second-trimester induced abortions, is the second leading cause of mortality in first-trimester induced abortions, and following infection.^[5]^ Recent studies have documented the continued importance of postabortion hemorrhage; a conservative estimate of the incidence of hemorrhage of clinical consideration is approximately 1% of procedures.^[6]^ A large study reported the incidence of hemorrhage requiring blood transfusions at 0.4%.

The administration of uterotonic agents, such as oxytocin, misoprostol, and methylergonovine, is recommended to prevent hemorrhage after induced abortion if the uterus is atonic.^[7,8]^ However, excessive use of oxytocin and misoprostol may cause high fever, shaking, chills, vomiting, hypertension, and other complications of toxicit.

Leonurus japonicus Houtt, commonly called Chinese motherwort Yi Mu Cao, is an herbaceous flowering plant native to Asia, and which has been used as medicinal herb for thousands of years in China.^[9,10]^ Pharmacological studies have shown that the active ingredients of motherwort injection (i.e., alkaloid, leonurine) could significantly facilitate hemostatic outcomes by promoting uterine contraction and blocking the uterine spiral vessels.^[11]^ Moreover, motherwort injection works on the lower uterus without the receptor saturation effect, which reduces the risk of adverse events caused by the excessive use of uterotonic agents. However, few studies have reported the use of motherwort in induced abortion. To provide reliable clinical evidence, we performed a protocol for systematic review and meta-analysis to evaluate the hemostatic effect of motherwort in postabortion.

## 2. Methods

This review protocol has been registered in the international prospective register of systematic reviews. Its registration number was CRD42022378022. The statement of preferred reporting items for systematic review and meta-analysis protocols ^[12]^ will be used as guidelines for reporting present review protocol. This study comes from published data, so no ethical approval is required.

### 2.1. Inclusion criteria

#### 2.1.1. Types of studies.

Original clinical randomized controlled trials (RCTs) assessing the beneficial effects and safety of motherwort on induced abortion will be included. There will be no restrictions on publication language or publication status.

#### 2.1.2. Types of participants

Pregnant women anticipating an induced abortion will be included. There will be no restrictions on the gender, age, or race of the participants.

#### 2.1.3. Types of interventions.

Intervention group receive motherwort injection given by any route of administration and dose used alone or in combination with oxytocin, while control group receive oxytocin alone or placebo.

#### 2.1.4. Types of outcomes.

The primary outcome was the volume of vaginal bleeding and its duration. Uterine size and dimension were measured as secondary endpoints. Blood coagulation indices, routine blood and adverse events were recorded to evaluate the safety.

### 2.2. Database search strategy

Computer retrieval and manual retrieval will be used to retrieve all the published literature independently by 2 authors. Databases searched include China National Knowledge Infrastructure, Chinese Scientific Journals Database, Wanfang Database, China Biological Medicine Database, PubMed, and EMBASE Database and Cochrane Central Register of Controlled Trials. All relevant RCTs will be collected from inception of each database to December 2022. The specific search strategy will be formulated with the specific database. We will search the reference lists of the relevant articles and will manually search Google Scholar to identify additional gray literature for inclusion. Table 1 shows the detailed search strategy in PubMed.

**Table 1:** Search strategy for the PubMed database.

#1 motherwort [Title/Abstract]
#2 leonurus [Title/Abstract]
#3 herba leonuri [Title/Abstract]
#4 Leonurus japonicus Houtt [Title/Abstract]
#5 leonurine [Title/Abstract]
#6 #1 OR #2 OR #3 OR #4 OR #5
#7 abortion [Title/Abstract]
#8 miscarry [Title/Abstract]
#9 termination of pregnancy [Title/Abstract]
#10 #7 OR #8 OR #9
#11 hemorrhage [Title/Abstract] #12 bleeding [Title/Abstract]
#13 blood loss [Title/Abstract]
#14 anemia [Title/Abstract]
#15 haemorrhagia [Title/Abstract]
#16 #11 OR #12 OR #13 OR #14 OR #15
#17 randomly [Title/Abstract]
#18 randomized [Title/Abstract]
#19 RCT [Title/Abstract]
#20 #17 OR #18 OR #19
#21 #6 AND #10 AND #16 AND #20

RCT = randomized controlled trial.

### 2.3. Study selection

**Figure 1.**
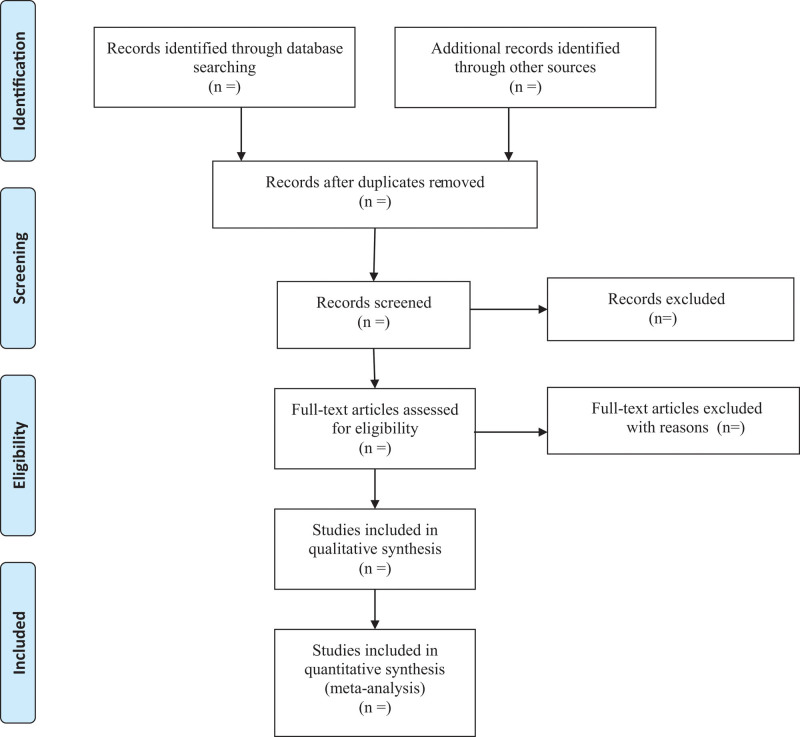
PRISMA flow chart of study selection process. PRISMA = Preferred Reporting Items for Systematic Review and Meta-Analysis.

First, 2 researchers will screen independently to identify titles and/or abstracts of studies that potentially meet the inclusion criteria. Second, those 2 researchers will independently assess the full texts of these potentially eligible studies for eligibility. Any disagreements between them will be resolved through discussion between them. The procedures of study selection will be performed in accordance with the preferred reporting items for systematic reviews and meta-analysis flow chart (as shown in Fig. 1).

### 2.4. Data extraction

A standardized, pre-defined, and pilot-tested form will then be used to extract data from the included studies for assessment of study quality and evidence synthesis. The extracted information will include the first author name, year of publication, country, sample size and dropout, details of participants, experimental intervention, comparison, duration of intervention, main outcome measures, adverse events, and information for assessment of the risk of bias (RoB). Two researchers will extract the data independently, any discrepancies will be identified and resolved through discussion. When the data are insufficient, ambiguous, or missing, and we will contact the corresponding authors of the original studies via e-mail.

### 2.5. Quality assessment

We will use the Cochrane collaboration tool to assess the RoB of included RCTs.^[13]^ Domains including random sequence generation, allocation concealment, blinding of participants, personnel, and outcome assessors, completeness of data outcome, selective reporting, and other biases. In case of disagreement, the third investigator was responsible for resolving it.

### 2.6. Statistical methods

All calculations were carried out with Stata 11.0 (The Cochrane Collaboration, Oxford, United Kingdom). Statistical heterogeneity was assessed based on the value of P and *I*^2^ using the standard chi-square test. When *I*^2^ > 50%, *P* < .1 was considered to be significant heterogeneous. The random-effect model was performed for meta-analysis; otherwise, the fixed-effect model was used. When possible, subgroup analyses were conducted to explore the origins of the heterogeneity. The results of dichotomous outcomes were expressed as risk difference with a 95% confidence intervals. For continuous various outcomes, mean difference or standard mean difference (SMD) with a 95% confidence intervals was applied. Sensitivity analyses to identify the robustness of the results of the meta-analysis will be conducted by excluding: studies with high RoB; and outliers that are numerically distant from the rest of the data.

### 2.7. Assessment of reporting biases

If there are more than 10 studies included in the meta-analysis, a funnel plot will be used to assess publication bias.^[14]^

## 3. Discussion

Unintended pregnancy is a problem that women encounter throughout their reproductive age. Around 1 in 4 women will experience an abortion in their lifetime, and about 55.7 million abortions take place worldwide every year.^[15]^ Excessive and prolonged uterine bleeding is one of the most common and critical adverse reactions of induced abortion, for it increases the risk of anemia and intrauterine infection.^[16,17]^ Therefore, reducing the uterine bleeding is very important to improve the satisfaction of induced abortion.

Chinese herbal medicine has been used to alleviate the postpartum hemorrhage in China for thousands years.^[18,19]^ Although traditional Chinese medicine etiology of abnormal uterine bleeding induced by medical abortion is similar to postpartum hemorrhage, there is still a lack of clinical evidence. Our systematic review will provide a detailed summary of the current evidence related to the efficacy of motherwort injection in preventing postabortion hemorrhage after induced abortion. This evidence will be useful to practitioners, patients, and health policy-makers regarding the use of motherwort injection after induced abortion.

## Author contributions

**Conceptualization:** Xiaohan Wang.

**Formal analysis:** Yongqian Gong.

**Writing – original draft:** Yanjie Xiang.

**Writing – review & editing:** Jianfeng Xiang.

## Correction

This article was originally published with Jianfeng Xiang’s name spelt incorrectly as Xianfeng Xiang. It has now been corrected in the online version.
